# Serological and molecular detection of *Toxoplasma gondii* and *Neospora caninum* in ruminants from Somalia

**DOI:** 10.1007/s00436-024-08397-6

**Published:** 2024-11-11

**Authors:** Monica T. A. Kakimori, Aamir M. Osman, Ana C. S. Silva, Abdalla M. Ibrahim, Mohamed A. Shair, Ana C. Cavallieri, Luiz D. Barros, João L. Garcia, Thállitha S. W. J. Vieira, Ahmed A. Hassan-Kadle, Rafael F. C. Vieira

**Affiliations:** 1 Graduate Program On Animal Science, Universidade Estadual de Londrina, Londrina, Paraná Brazil; 2 Graduate Program On Veterinary Sciences, Universidade Federal Do Paraná, Curitiba, Paraná Brazil; 3 Somali One Health Centre, Abrar University, Muqdisho, Somalia; 4 Department of Animal Health and Veterinary Services, Ministry of Livestock, Forestry, and Range, Mogadishu, Somalia; 5 Center for Computational Intelligence to Predict Health and Environmental Risks (CIPHER), The University of North Carolina at Charlotte, Charlotte, USA; 6 Abrar Research and Training Centre, Abrar University, Mogadishu, Somalia; 7 Department of Veterinary Medicine, Universidade Federal de Lavras, Lavras, Minas Gerais Brazil; 8 Department of Chemistry, The University of North Carolina at Charlotte, Charlotte, USA; 9 Department of Epidemiology and Community Health, The University of North Carolina at Charlotte, Charlotte, USA

**Keywords:** *Toxoplasma gondii*, *Neospora caninum*, Ruminants, Zoonotic disease, Somalia, Sub-Saharan Africa

## Abstract

*Toxoplasma gondii* and *Neospora caninum* infect a wide range of domestic and wild animals, including humans, in the case of *T. gondii*, and cause economic losses in livestock due to abortion and neonatal mortality. In Somalia, zoonotic diseases are concerning due to cultural practices and livestock’s economic importance, but surveillance is limited. This study aimed to determine the seroprevalence and molecular prevalence of *T. gondii* and *N. caninum* in Somali sheep, goats, and cattle. A cross-sectional study was conducted between December 2018 and January 2020 in Benadir and Lower Shabelle regions of Somalia. Blood samples were collected from 128 cattle, 184 goats, and 46 sheep. Serum samples were tested for anti-*T. gondii* and anti-*N. caninum* antibodies using IFAT, and PCR was performed on extracted DNA to detect *T. gondii* and *N. caninum* DNA. Overall, 106/358 (29.6%) animals tested positive for anti-*T. gondii* antibodies, with the highest prevalence in sheep (62.5%), followed by goats (30.4%) and cattle (15.6%) (*P* < 0.001). For anti-*N. caninum* antibodies, 13/358 (3.6%) animals tested positive, with cattle showing the highest prevalence (6.2%), followed by goats and sheep (both 2.2%). Co-seropositivity for both antibodies was found in cattle and sheep. Molecular detection of *T. gondii* DNA revealed a prevalence of 9/358 (2.5%), primarily in sheep (15.2%) and cattle at 1.6% while all goat samples tested negative. No samples were positive for the *N. caninum* Nc5 gene. This study reveals *T. gondii* and *N. caninum* prevalence in Somali ruminants, highlighting the need for better surveillance and control.

## Introduction

The phylum Apicomplexa constitutes a diverse group of parasitic protists, comprising over 6000 species (Votýpka et al. 2016; Rojas-Pirela et al. 2021), which inhabits various environments, including soil, freshwater, and marine habitats (del Campo et al. 2019). Apicomplexan parasites are responsible for a variety of diseases in humans and animals, from vector-borne diseases such as malaria (*Plasmodium* spp.), babesiosis (*Babesia* spp.), theileriosis (*Theileria* spp.) to orally transmitted parasites, such as toxoplasmosis (*Toxoplasma gondii*), neosporosis (*Neospora* spp.), and cryptosporidiosis (*Cryptosporidium* spp.) (Rojas-Pirela et al. 2021). Many of these parasites have significant clinical and economic implications, as they are responsible for important human and veterinary diseases worldwide (Müller and Hemphill 2013).

*Toxoplasma gondii* and *Neospora caninum* are intracellular protozoan parasites that infect a wide range of domestic and wild animals. While *T. gondii* is zoonotic and can infect humans, *N. caninum* is not zoonotic and does not pose a direct risk to human health (King et al. 2010; Dubey 2009). Both parasites have complex life cycles involving multiple hosts. Some canines serve as definitive hosts for *N. caninum* (dogs, coyotes, gray wolves, and dingoes), while felids acting as definitive hosts for *T. gondii* (Dubey and Schares 2011; Hill and Dubey 2002). Ruminants, on the other hand, serve as intermediate hosts and become infected by ingesting pastures, food, or water, contaminated with sporulated oocysts, or vertically, by transplacental transmission from the dam to the fetus (Dubey 2009, 2003). These parasites are significant causes of reproductive disorders and economic losses in livestock production (Basso et al. 2022).

Toxoplasmosis is widely recognized as one of the most prevalent parasitic diseases affecting both humans and animals. It is estimated that *T. gondii* infects up to one-third of the global human population (Jones and Dubey 2010). Neosporosis is a significant contributor to infectious abortion in cattle worldwide, as well as in small ruminants (Dubey and Schares 2011; de Barros et al. 2021; de Souza et al. 2022; Macedo et al. 2017). In ruminants, primary maternal infections cause toxoplasmosis and neosporosis, leading to various adverse outcomes, including embryonic death, fetal death, abortion, stillbirth, and neonatal death. Notably, toxoplasmosis tends to be more severe in goats compared to sheep, while cattle and water buffaloes exhibit greater resistance to acute clinical toxoplasmosis compared to other livestock species (de Freitas Silva Filho et al. 2008; Lindsay and Dubey 2020, de Barros et al. 2020).

In Africa, both *T. gondii* and *N. caninum* infections have been documented in various wildlife and domestic animals. *T. gondii* has been reported in humans, cats, sheep, goats, cattle, camels, lions, birds, cheetahs, and leopards (Yusuf et al. 2021; Lobetti and Lappin 2012; Hammond-Aryee et al. 2015; Cheadle et al. 1999; Tonouhewa et al. 2019; Khames et al. 2018; Lukášová et al. 2018; Penzhorn et al. 2002), while N*. caninum* infections have been observed in cattle from Algeria (Ghalmi et al. 2009), Egypt (Ibrahim et al. 2009), and Kenya (Okumu et al. 2019), camel, and ruminats from Sudan (Ibrahim et al. 2015), dogs from Algeria (Ghalmi et al. 2009), and zebra (*Equus burchelli*), eland (*Taurotragus oryx*), African buffalo (*Syncerus caffer*), Thompson gazelle (*Gazella thompsoni*), impala (*Aepyceros melampus*), warthog (*Phacochoerus aethiopicus*), spotted hyena (*Crocuta crocuta*), and in free-ranging cheetah (*Acinonyx jubatus*) from Kenya (Ferroglio et al. 2003).

In Somalia, recent studies show a concerning picture. High prevalence rates of *anti-T. gondii* antibodies among pregnant women, with rates of 45.2% and 51.8% reported by Hassan et al. (2023) and Yusuf et al. (2021) respectively, suggesting potential exposure to the parasite. Similarly, livestock in Somalia has shown significant infection rates, with Hassan-Kadle (2014) reporting prevalence rates of 6.3% in camels, 7.1% in cattle, 34.5% in sheep, and 26.7% in goats. However, there is a lack of information on *N. caninum*, another parasite affecting animals. These findings raise concerns, but Somalia lacks robust efforts to detect and control these zoonotic diseases. Cultural practices like consuming unpasteurized milk and handling animal products without gloves further heighten the risk of zoonotic diseases at the human-animal interface within Somali communities (Hassan-Kadle et al. 2021). Additionally, while *N. caninum* has not been demonstrated as a zoonosis, it remains a significant contributor to infectious abortion in cattle worldwide, as well as in small ruminants (Dubey and Schares 2011). Given the critical role livestock plays in Somalia’s economy and food security, this study aims to detect both *T. gondii* and *N. caninum* in Somali ruminants using serological and molecular methods. This improved surveillance will contribute to a more comprehensive understanding of parasitic diseases in livestock and inform the development of effective control strategies to protect both animal and human health.

## Material and methods

### Study design

**Fig. 1:**
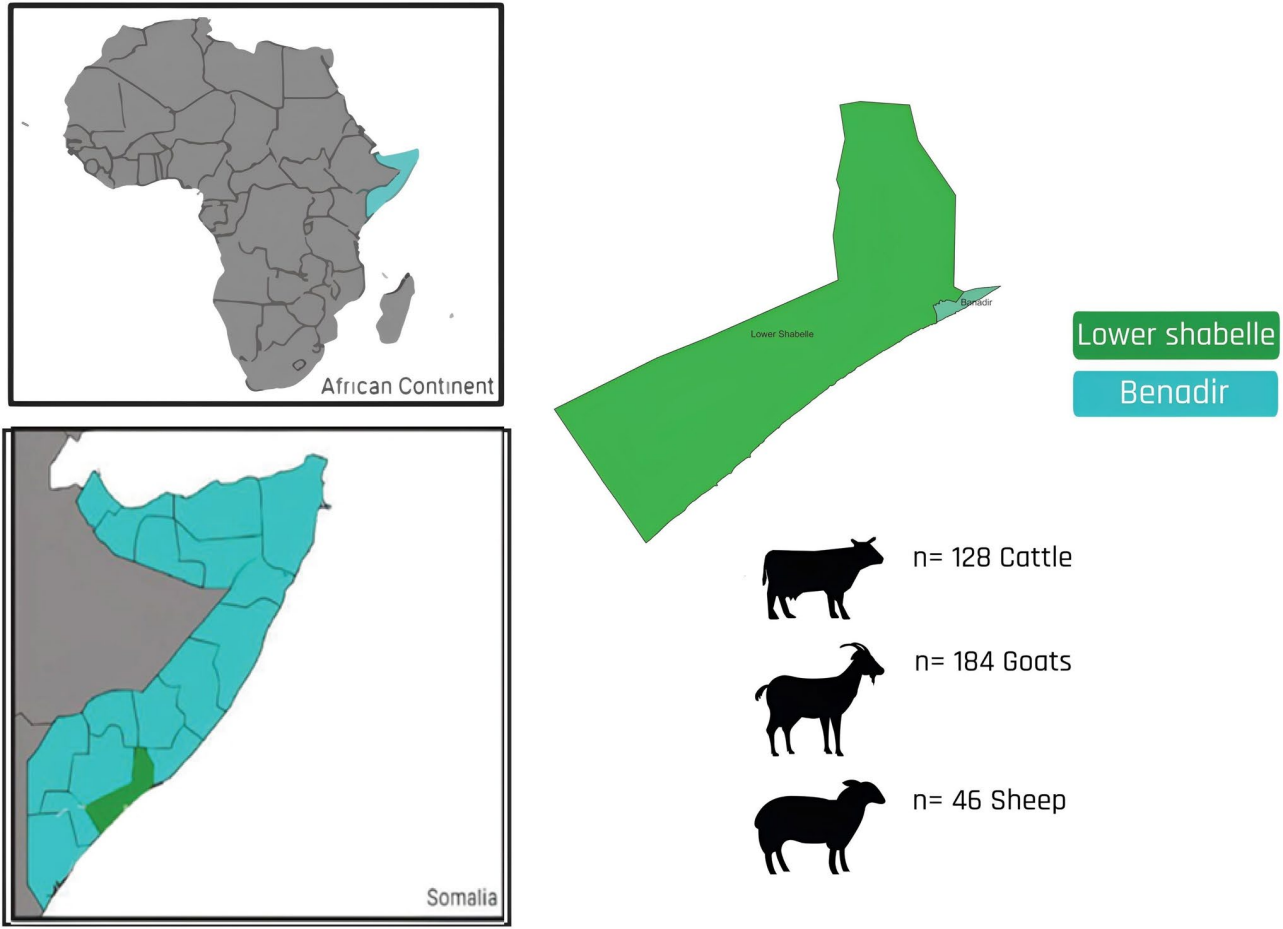
Map of Somalia showing the sampling sites and number of animals sampled. The highlighted blue (Benadir region) and green (lower Shabelle region) indicate the locations of the sampled area. The figure was generated and modified using QGIS software version 3.26.0

A cross-sectional study was carried out between December 2018 and January 2020 on Benadir (2.1065° N, 45.3933° E) and Lower Shabelle Region (1.8670° N, 44.5502° E), Somalia (Fig. 1).

A non-probabilistic convenience sampling was performed. A total of 128 cattle, 184 goats, and 46 sheep blood samples were previously surveyed for *Bartonella* (Osman et al. 2024). Five milliliters of blood were placed into tubes containing a serum separator gel (BD Vacutainer® Franklin Lakes, NJ, USA) and kept at room temperature 25 °C until visible clot formation. The samples were centrifuged at 1500 × g for 5 min, serum separated, and kept at − 20 °C for serological testing.

### Serological analysis

Serum samples were used to detect anti-*T. gondii* and *anti-N. caninum* antibodies by indirect fluorescence antibody test (IFAT) as previously described by Camargo (1973) and Paré et al. (1995), respectively. Tachyzoites of *N. caninum* Nc-1strain and *T. gondii* RH strain previously cultivated in Vero cells were used as crude antigens. Seropositive control serum samples obtained from naturally infected cattle, goats, and sheep with *N. caninum* and *T. gondii* (de Freitas Silva Filho et al. 2008; de Barros et al 2021) and non-reactive serum (negative control) were included in all slides.

Conjugates were species-specific anti-IgG antibodies for cattle, goats, and sheep (Sigma-Aldrich, St. Louis, MO, USA). Serum samples that showed fluorescence through all parasite contours and titers ≥ 64 for *T. gondii* to all species (Rodrigues et al. 2021; Silva et al. 2021; Bernardes et al. 2021), ≥ 50 for *N. caninum* in sheep and goats (Novoa et al. 2023), and ≥ 100 in cattle (Fávero et al. 2017) were considered positive. Endpoint titers were determined to be the higher dilution in which peripheral fluorescence was visualized around the parasite.

### DNA extraction and PCR

DNA was extracted from dried blood spots (DBS) on filter paper and from 200 μL of EDTA-blood samples using a commercial kit (PureLink™ genomic DNA kit, Thermo Fisher Scientific, Waltham, MA, USA) according to the manufacturer’s instructions. A conventional PCR for the mammalian endogenous gene glyceraldehyde-3-phosphate dehydrogenase (*gapdh*) was performed in all samples to monitor DNA extraction (Birkenheuer et al. 2003). The amplification of *T. gondii* DNA was performed using the primers TOX4 (CGCTGCAGGGAGGAAGACGAAAGTTG) and TOX5 (CGCTGCAGACACAGTGCATCTGGATT) aiming at a repetitive fragment of 529 bp in the genome of *T. gondii* (Homan et al. 2000). DNA from tachyzoites of RH strain and sterile ultrapure water was used as positive and negative controls, respectively. PCR reactions contained by 1X PCR buffer, 0.2 mM of each dNTP, 2 mM MgCl_2_, 0.5 U of Taq polymerase (Platinum Taq DNA Polymerase, Thermo Fisher Scientific), 1.0 μM of each primer, and 5 μL of genomic DNA in a final volume of 25 μL. Amplification reactions were performed in an automatic DNA thermal cycler (Veriti Thermal Cycler; Applied Biosystems, Waltham, MA, USA) under the following conditions: 94 °C for 5 min for initial denaturation; 35 cycles of 94 °C for 60 s, 55 °C for 60 s, and 72 °C for 60 s; and a final extension step at 72 °C for 10 min.

For the detection of *N. caninum* DNA, the PCR was performed using the primers Np21 plus (5′-GGGTGTGCGTCCAATCCTGTAAC-3′3′) and Np6 plus (5′-CTCGCCAGTCAACCTACGTCTTCT-3′3′) which amplify a fragment of 337 bp of the Nc5 gene (Liddell et al., 1999). PCR reaction contained 2 μL of genomic DNA, 1 × PCR buffer, 0.2 mM of each dNTP, 2.5 mM MgCl_2_,1.25 U of Taq polymerase (Platinum Taq DNA Polymerase, Invitrogen, USA), and 0.8 μM of each primer in a final solution of 25 μL. Reactions were carried out in an automatic DNA thermal cycler (Veriti Thermal Cycler, Applied Biosystems) following the conditions: 94 °C for 5 min for initial denaturation, 35 cycles of 94 °C for 30 s, 63 °C for 30 s, 72 °C for 1 min followed by a final extension step at 72 °C for 7 min. Positive and negative controls, constituted of DNA from tachyzoites of Nc-1 strain and ultrapure water, respectively, were included in all reactions.

After the PCR reaction, all samples were viewed using electrophoresis in 1.5% agarose gel with SYBR Safe DNA Gel Stain (Thermo Fisher Scientific) and visualized under UV light.

### Statistical analysis

Data analyses were performed with SPSS Statistics software® (IBM® Corp, Armonk, NY, USA, version 26). The chi-square test was used to evaluate significant differences in infection rates of different animal species. Odds ratio (OR), 95% confidence intervals (95% CI), and *P*-values were calculated separately for each variable, and results were considered significant when *P* ≤ 0.05. Data were compiled and analyzed in Epi Info™ software, version 7.2.3.1 (Centers for Disease Control and Prevention, CDC, USA).

## Results

Overall, 106/358 (29.6%, 95% CI 24.9–34.6%) of animals tested positive for anti-*T. gondii* antibodies. Higher occurrence was observed in sheep 30/46 (62.5%, 95% CI 46.8–78.7%) (endpoint titer 64–1,048,576), followed by goats 56/184 (30.4%, 95% CI 23.9–37.6%) (endpoint titer 64–65,536), and cattle 20/128 (15.6%, 95% CI 9.8–23.1%) (endpoint titer 64–4096). Sheep (OR 10.1, 95% CI 4.7–21.9, *P* < 0.001, *χ*^2^ = 40.6), and goat (OR 2.4, 95% CI 1.3–4.2, *P* < 0.001, *χ*^2^ = 8.9), were more likely to have anti-*T. gondii* antibodies than cattle.

Overall, 13/358 (3.6%, 95% CI 1.9–6.1%) of the animals tested positive for anti-*N. caninum* antibodies. According to each species, higher occurrence was observed in cattle 8/128 (6.2%, 95% CI 2.7–11.9%) (endpoint titer 100–800), followed by goats 4/184 (2.2%, 95% CI 0.6–5.5%) (endpoint titer: 50–100), and sheep 1/46 (2.2%, 95% CI 0.1–11.8%) (endpoint titer 50). Cattle (OR 3, 95% CI 0.3–24.6, *P* = 0.51, *χ*^2^ = 1.1), and goat (OR 1, 95% CI 0.1–9.2, *P* > 0.5, *χ*^2^ = 0), were more likely infected by by presenting antibodies *N. caninum* than sheep; however, these differences were not statistically significant.

At the animal level, co-seropositivity for anti-*T. gondii* and anti-*N. caninum* were detected in 3 of 358 animals (0.84%; 95% CI 0.2–2.4%). This co-seropositivity was observed in two cattle and one sheep, while no co-seropositivity was found in goats. The *gapdh* gene was consistently amplified from all DNA blood samples. Overall, 9/358(2.5%, 95% CI 1.1–4.6%) of animals were positive for *Toxoplasma* PCR. A higher molecular detection rate was observed in sheep 7/46 (15.2%, 95% CI 6.6–30.1%), followed by cattle 2/128 (1.6%, 95% CI 0.2–5.4%). All goat DNA samples tested negative for *Toxoplasma*. All PCR-positive animals also had detectable anti-*T. gondii* antibodies. Regarding the *N. caninum* Nc5 gene, none of the samples showed positivity.

Multiple attempts to sequence of the detected *T. gondii*, by the Sanger method, failed due to the occurrence of faint in the agarose gel electrophoresis. Unfortunately, attempts to clone the PCR amplicon also failed.

## Discussion

This study reports, for the first time, *T. gondii* and *N. caninum* in ruminants, 106/358 (29.6%) and 13/358 (3.6%), respectively, using combined serology and molecular techniques from Somalia. Previous studies conducted in goats and sheep from Switzerland also reported higher prevalence rates of *T. gondii* (77.1%) and comparable prevalence rates of of *N. caninum* (1.2%) (Basso et al. 2022). Another study from Argentina showed comparable prevalence rates of (32.6%) *T. gondii* and higher prevalence rates of (14.9%) *N. caninum* (Novoa et al. 2023). In contrast, studies conducted in China show lower prevalence rates of 21.33% *T. gondii* and 8.7% *N. caninum* (Liu et al. 2015). These variations in prevalence rates may be attributed to differences in geographical locations, environmental factors, management practices, and diagnostic techniques used across regions.

*Toxoplasma gondii* and *N. caninum* are closely related intracellular protozoan parasites known to cause significant disease and economic losses in the farming industry. *Toxoplasma gondii* primarily induces abortion and fetal abnormalities in sheep, whereas *N. caninum* is associated with similar outcomes in cattle (Dubey 2009, 2003). In our study, we observed a higher occurrence of anti-*T. gondii* antibodies in sheep (62.5%), followed by goats (30.4%) and cattle (15.6%). Conversely, a higher occurrence of *N. caninum* antibodies was observed in cattle (6.2%), followed by goats (2.2%) and sheep (2.2%). These findings align with the notion that neosporosis is considered a more significant contributor to reproductive problems in cattle, while toxoplasmosis is more prevalent in sheep (de Freitas Silva Filho et al. 2008; Lindsay and Dubey 2020, de Barros et al. 2020).

In our study, the observed prevalence of *T. gondii* infection in cattle, at 15.6%, exceeds rates reported in several other regions, such as Tunisia (5%) (Amdouni et al. 2021), Brazil (2%) (Santos et al. 2010), and Switzerland (3.8%) (Berger-schoch et al. 2011). Conversely, it falls below rates documented in Colombia (36.3%) (Franco-Hernandez et al. 2016) and Northern Portugal (50%) (Lopes et al. 2015), but is comparable to rates reported in Tunisia (19.3%) (Amdouni et al. 2017). Conversely, the prevalence of *N. caninum* infection in our study, at 6.2%, is notably lower compared to rates reported in Kenya (26.0%) (Okumu et al. 2019), Algeria (19.64%) (Ghalmi et al. 2009), and Senegal (17.9%) (Kamga-Waladjo et al. 2010). Moreover, higher seroprevalences of *N. caninum* compared to *T. gondii* in cattle were also observed in other studies, such as those reported in Tunisia (Amdouni et al. 2021), inconsistent with our findings.

Previous studies have reported cross-reactions of *T. gondii* and *N. caninum* antibodies (Huertas-López et al. 2021; Sánchez-Sánchez et al. 2021). However, this issue appears to be irrelevant in our study, as only a very low proportion of animals tested positive for *N. caninum* antibodies. Additionally, we observed co-seropositivity in cattle and sheep but not in goats, suggesting that potential cross-reactions were not significant. These findings are consistent with a study conducted in Switzerland (Basso et al. 2022).

In epidemiological studies, serology plays a crucial role in detecting antibodies against *T. gondii* and *N. caninum*, primarily using the IFAT, known for its high specificity despite potential cross-reactions (Dubey et al. 1988; Dubey and Lindsay 1996; Abdelbaky et al. 2020). Additionally, the enzyme-linked immunosorbent assay (ELISA) is commonly employed for *T. gondii* and *N. caninum* detection in ruminants due to its easily use (Fortes et al. 2018; Iovu et al. 2012). However, IFAT remains the gold standard in serodiagnostics for these infections. In our study, IFAT was utilized alongside molecular detection through PCR. Notably, this study marks the first investigation of *T. gondii* and *N. caninum* in the region. Overall, 9 out of 358 animals (2.5%) tested positive for *T. gondii* by PCR. Regarding *N. caninum* detection using the Nc5 gene, none of the samples yielded positive results.

The discrepancy between the number of animals testing positive for *T. gondii* and *N. caninum* antibodies compared to those testing positive in PCR assays is noteworthy. Previous research has demonstrated that the IFAT exhibits a sensitivity of 80.4% and specificity of 91.4% for detecting *T. gondii* antibodies (Shaapan et al. 2008), while detecting *N. caninum* antibodies has a sensitivity of 98% and specificity of 99% (Packham et al. 1998). The lower number of positive animals in PCR assays may be attributed to the potential presence of low parasite loads in the bloodstream, which could evade detection by the PCR protocols utilized in our study.

The findings of this study indicate the presence of *T. gondii* infection in studied animals, suggesting environmental contamination with *T. gondii* oocysts and handling of aborted materials and reproductive excretions with bare hands. This poses a potential risk for human infection with *T. gondii*, as humans can acquire the infection by ingesting contaminated food, water, or undercooked meat (Dubey 2009, 2003). Although meat and milk from cattle, sheep, and goats were not tested in this study, previous research has demonstrated the isolation of viable *T. gondii* from meat and milk (de Macedo et al. 2012; Dubey et al. 2011; Fusco et al. 2007), indicating that ruminants could serve as a source of *T. gondii* infection for humans. Therefore, further investigations are warranted to assess whether meat and milk from these or other farms in the province are contaminated.

The limitation of this study is the lack of information on the pregnancy status of the sampled animals, which is crucial since *T. gondii* and *N. caninum* are well-documented causes of abortion in ruminants. This limits our ability to fully evaluate the impact of these pathogens on reproductive health. However, the study still provides valuable baseline prevalence data that can guide future research on reproductive losses. Additionally, performing PCR on blood samples presents a challenge, as *Toxoplasma* and *Neospora* are typically present in the blood only during acute infection or recrudescence, making it unlikely to detect DNA in chronically infected animals. Despite these limitations, the detection of antibodies through serological analysis offers a broader understanding of both past and recent exposures, providing valuable baseline prevalence data.

In conclusion, this study provides valuable insights into the prevalence of *T. gondii* and *N. caninum* infections among ruminants in Somalia. Our findings highlight the significant presence of *T. gondii* antibodies in sheep, goats, and cattle with potential implications for human health due to environmental contamination. Furthermore, the detection of *N. caninum* antibodies in ruminants underscores the importance of addressing reproductive issues in livestock. Additionally, further research is warranted to explore the sources and routes of transmission of these parasites and to implement effective preventive strategies. By enhancing our understanding of the epidemiology of *T. gondii* and *N. caninum* infections in Somalia, we can take proactive steps toward promoting animal welfare, ensuring food safety, and protecting public health in the region.

## Data Availability

No datasets were generated or analysed during the current study.
